# A clinical trial of a unidirectional porous tricalcium phosphate filling for defects after resection of benign bone lesions: a prospective multicenter study

**DOI:** 10.1038/s41598-022-20359-5

**Published:** 2022-09-26

**Authors:** Kunihiro Ikuta, Yoshihiro Nishida, Takehiro Ota, Satoshi Tsukushi, Eiji Kozawa, Hiroatsu Nakashima, Kenji Yamada, Satoshi Yamashita, Shiro Imagama

**Affiliations:** 1 Department of Orthopaedic Surgery, Nagoya University Graduate School and School of Medicine, 65 Tsurumai, Showa, Nagoya, Aichi 466-8550 Japan; 2 Department of Rehabilitation, Nagoya University Hospital, 65 Tsurumai, Showa, Nagoya, Aichi 466-8550 Japan; 3 Department of Orthopaedic Surgery, Aichi Cancer Center Hospital, 1-1, Kanokoden, Chikusa, Nagoya, Aichi 464-8681 Japan; 4 Department of Orthopaedic Surgery, Nagoya Memorial Hospital, 305-4, Hirabari, Tempaku, Nagoya, Aichi 468-8520 Japan; 5 Department of Orthopaedic Surgery, Gifu Prefectural Tajimi Hospital, 161-5, Maehata, , Tajimi, Gifu 507-8522 Japan; 6 Department of Orthopaedic Oncology, Okazaki City Hospital, 3-1, Goshoai, Koryuji, Okazaki, Aichi 444-8553 Japan; 7 Medical IT Center, Nagoya University Hospital, 65 Tsurumai, Showa, Nagoya, Aichi 466-8550 Japan

**Keywords:** Medical research, Oncology, Rheumatology

## Abstract

Affinos (Kuraray, Japan) is a β-tricalcium phosphate bone substitute with a unidirectional porous structure. This study aimed to investigate its efficacy on the healing process after filling for bone defects. Fifty-six patients who met the inclusion criteria were divided into cohort 1 (n = 30), including bones other than phalanges and metacarpal/tarsal bones, and cohort 2 (n = 26), including phalanges and metacarpal/tarsal bones. Semi-quantified scores for material resorption and trabeculation through the defect were evaluated with radiographs after surgery. In some patients, levels of bone metabolic markers were assessed. The values of resorption and trabeculation increased steadily with time, and trabeculation progressed compared with resorption in both cohorts. In cohort 1, multiple regression analyses showed that the diaphyseal lesion, smaller defect volume, and increased resorption values at 3 months were associated with increased values of resorption 12 months after surgery (R^2^ = 0.66, p < 0.001). The trabeculation values at 2 months were positively related to the trabeculation values 12 months after surgery (R^2^ = 0.35, p = 0.002). In cohort 2, the increased resorption values at 2 months and smaller defect volume significantly correlated with the increased resorption values 12 months after surgery (R^2^ = 0.58, p < 0.001). The ratio from the baseline of pyridinoline cross-linked carboxyterminal telopeptide of type I collagen at 3 months was negatively associated with the trabeculation values 12 months after surgery (R = − 0.791, p = 0.004). Evaluation of radiographic images and bone metabolic markers in the early postoperative period may predict the healing status at 12 months postoperatively in the defects followed by Affinos filling.

## Introduction

After the surgical procedure for benign bone lesions, use of synthetic bone substitutes is currently a standard treatment for bone defects. Hydroxyapatite (HA) or β-tricalcium phosphate (β-TCP) has been widely used to maintain the structural and functional stability of the bone and adjacent joint. However, these synthetic bone substitutes have mostly osteoconductive characteristics but no osteoinductive capacity. Although HA is useful for supporting the surrounding bone due to its initial mechanical strength, it has the disadvantage of interference with remodeling to the normal bone^1,2^. On the other hand, β-TCP is characterized by its ability to be incorporated into the surrounding bone^1,2^. However, although its high porosity can contribute to bone regeneration, concerns have been raised that β-TCP is weak in strength and will eventually break down^1–3^.

Affinos (Kuraray, Japan) was developed as a β-TCP material with low porosity and relatively high compressive resistance. The porosity of Affinos is 57 ± 5%, and it has a unique structure in which many interconnected pores (pore size: 25–300 μm) are arranged in a single direction, facilitating the ingrowth of blood vessels and bone tissue.

In directions parallel and perpendicular to the pores, the initial compressive strength is 8 MPa and 1.5 MPa, respectively. In vivo, it has shown a high osteoconductive ability to induce bone regeneration due to its unidirectionally oriented pores^4^. β-TCP with a spherical interconnected porous structure is commonly used as a suitable bone-filling material. Murayama et al. reported that angiogenesis was increased within Affinos compared to β-TCP with a spherical interconnected porous structure in a vascularized pedicle rat model^5^. However, there has been a lack of clinical information on the regeneration process of bone defects filled with Affinos^6^.

Bone regeneration in bone defects filled with bone substitutes is primarily assessed by radiological examination, but it may be helpful to combine other modalities, possibly including quantitative measurements. Identifying bone metabolic markers to monitor the effects of treatment would facilitate patient management. However, few studies have investigated this issue in synthetic bone substitutes^7,8^.

The primary objective of this study was to evaluate radiographically the healing process of bone defects filled with Affinos in patients treated with resection of benign bone lesions. Second, we sought to determine the variables affecting the healing status of the bone defect 12 months after surgery in these patients. In addition, we analyzed changes in bone metabolic markers as related to the healing status 12 months after surgery in patients treated at Nagoya University Hospital.

## Patients and methods

### Patients

This study’s protocol was approved by the ethics committee of Nagoya University Graduate School and School of Medicine (No. 2016-0122) and the institutional review board of each institution. Written informed consent was obtained from all patients. All research was performed in accordance with relevant guidelines/regulations, and the Declaration of Helsinki. We prospectively enrolled 74 patients with benign bone tumors or tumor-like lesions for whom a surgical procedure was planned between July 2017 and March 2020. The inclusion criteria were as follows: (1) benign bone tumor or bone tumor-like lesion indicated for surgical treatment, (2) intralesional curettage or *en bloc* resection followed by filling with Affinos as a bone substitute, (3) availability for radiographs at scheduled time points (preoperative, immediately postoperative, and 1, 2, 3, 6, 9, and 12 months postoperative) and (4) consent for our study. The exclusion criteria were as follows: (1) history of previous treatment with drugs including bisphosphonates, denosumab, and steroids, (2) use of other materials in addition to Affinos during the procedure.

**Figure 1:**
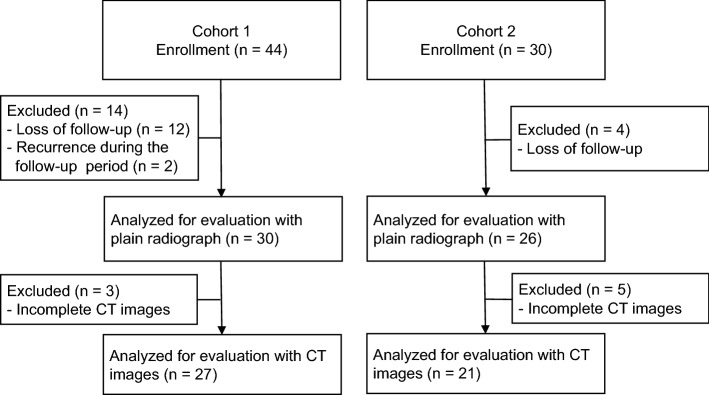
Flowchart of the study patients. *CT* computed tomography.

On account of not achieving a follow-up of 12 months after surgery, 16 patients were excluded. Two giant cell tumors of bone recurred during the follow-up period of 12 months: one was in the forearm 8 months after surgery and the other in the proximal fibula 9 months after surgery (Fig. 1). These were not included due to insufficient data and pathological findings. Eventually, 56 patients were eligible and analyzed in this study. Considering the significant small lesions in the fingers and toes compared with those in other bones, the process of bone regeneration in defects filled with Affinos was expected to differ between these bones. Thus, we divided patients into cohort 1 (any bones other than other than phalanges and metacarpal/tarsal bones) and cohort 2 (phalanges and metacarpal/tarsal bones) according to the affected bones, and analyzed the outcomes separately. All procedures described in this study were performed in compliance with the ethical standards set by the Helsinki Declaration of 1975 (revised in 2000) and the regulation of national laws.

### Treatment and assessment

Musculoskeletal oncologists performed surgical procedures at each institution. After curettage of the lesion, the defect was loosely filled with Affinos so as not to break its porotic structure. The surgeons individually decided whether to use granular and/or block types for bone defects. They aligned the pore structure of the blocks with the principal strain axis that was experienced in the defect. Prophylactic fixation with locking plates was used in 2 patients with a tibial lesion. En bloc excision was performed in patients with osteoid osteoma. Pathological diagnosis was confirmed by each institutional pathologist. A gradual increase in weight-bearing was allowed during the follow-up period depending on the patient’s symptoms and the radiographic findings. Range of motion exercises was started immediately after surgery.

### Radiological evaluation of healing status

**Figure 2:**
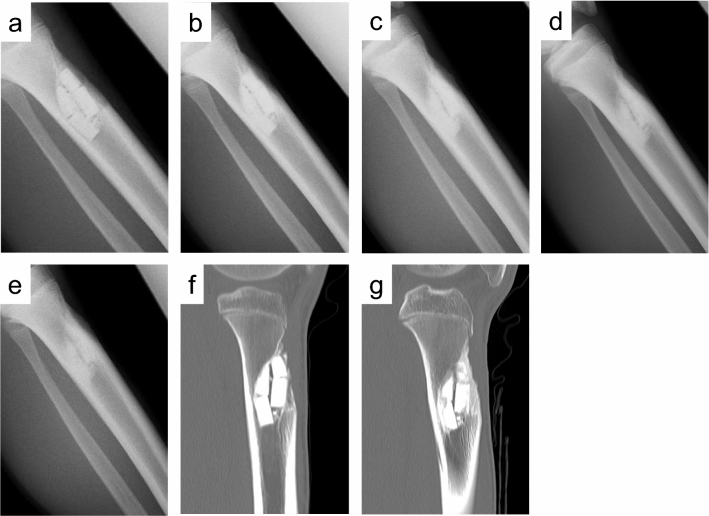
The degrees of Affinos filling the defects were classified into five stages with plain radiographs and computed tomography (CT) images in a 13-year-old boy with tibial fibrous dysplasia. (**a**) Radiograph at 1 month after surgery: the values of resorption and trabeculation were 0% and 0%, respectively. (**b**) Radiograph at 3 months after surgery: the values of resorption and trabeculation were 25% and 50%, respectively. (**c**) Radiograph at 6 months after surgery: the values of resorption and trabeculation were 75% and 75%, respectively. (**d**) Radiograph at 9 months after surgery: the values of resorption and trabeculation were 75% and 100%, respectively. (**e**) Radiograph at 12 months after surgery: the values of resorption and trabeculation were 100% and 100%, respectively. (**f**) Sagittal view of tibial CT at 3 months after surgery: the values of resorption and trabeculation were 25% and 50%, respectively. (**g**) Sagittal view of tibial CT at 9 months after surgery: the values of resorption and trabeculation were 50% and 75%, respectively.

Patients underwent evaluation with plain radiographs taken at scheduled time points (preoperative, immediately postoperative, and at 1, 2, 3, 6, 9, and 12 months postoperative). The volume of bone defect was calculated as the product of the anteroposterior, transverse, and cephalocaudal dimensions of a lesion measured on radiographs immediately after surgery^9^. The cephalocaudal length was measured as the maximum diameter orthogonal to the anteroposterior and transverse dimensions. In order to assess the regeneration process of Affinos in bone defects with radiographic examinations, a semi-quantitative classification was applied based on the five-stage evaluation system of filling materials reported by Anker et al.^9,10^. Resorption of Affinos and bone trabeculation through the defect were scored as 0%, 25%, 50%, 75%, or 100%. Two independent reviewers (KI and TO), both experienced orthopedic surgeons, evaluated the radiographs. When agreement could not be reached, the score was decided by discussion. Interobserver concordance was assessed by kappa statistic (κ). The degree of agreement for κ-value was interpreted as follows: lower than 0.20, poor; 0.21–0.40, fair; 0.41–0.60, moderate;0.61–0.80, good; and 0.81–1.00, excellent. Patients underwent computed tomography (CT) at 3 and 9 months postoperatively as references for rating the values with plain radiographs. Figure 2 showed an example of assessment of plain radiographs and CT images in determining the values of resorption and trabeculation.

### Bone metabolic markers

To investigate the clinical value of bone metabolic markers regarding the clinical course of healing, we harvested blood samples from 15 patients available for consent. To ensure reliability of blood samples, marker measurements were performed on patients treated at our hospital (Nagoya University Hospital). The levels of bone metabolic markers were measured at baseline (the day after surgery) and 3 and 6 months after surgery. The bone metabolic markers included pyridinoline cross-linked carboxyterminal telopeptide of type I collagen (1CTP) and tartrate-resistant acid phosphatase 5b (TRACP-5b) as bone resorption markers and procollagen type 1 amino-terminal propeptide (P1NP) and bone-specific alkaline phosphatase (BAP) as bone formation markers. Serum TRACP-5b, BAP, 1CTP, and P1NP were measured by SRL Inc. (Tokyo, Japan). The serum level of TRACP-5b was determined with an enzyme immunoassay using the Osteolinks TRAP-5b kit (Nittobo Medical, Japan). Serum BAP was measured with a chemiluminescence enzyme immunoassay method using the Beckman Access Ostase assay kit with a UniCel DxI 800 system (Beckman Coulter K.K., Japan). For the 1CTP assay, the double antibody radioimmunoassay method using a kit provided by Orion Diagnostica (Espoo, Finland) was used, and radioactivity was determined with a γ-counter (ARC950; Hitachi). The double antibody sandwich method was used to measure P1NP with electrochemiluminescence immunoassay using Elecsys total P1NP (Roche Diagnostics, Germany).

### Analysis

Statistical analyses were performed using Student t-test for continuous variables. Variables based on repeated observations were compared using repeated measures ANOVA. To evaluate factors associated with the healing status 12 months after surgery, the clinical variables including defect volume, affected bones, gender, the location within the bone (diaphysis, metaphysis, and epiphysis), types of the lesion (cystic vs. solid), and age, as well as radiographical assessments of the values of resorption and trabeculation up to 3 months postoperatively were examined by multiple regression analysis. For categorical variables, multiple regression analysis was conducted with dummy variables. Spearman rank method was used to analyze correlations between the levels of bone metabolic markers and the healing status 12 months after surgery. P-values less than 0.05 were considered statistically significant. SPSS 27.0 for Windows software (SPSS, Inc., Chicago, IL, USA) was used for the statistical analyses.

### Ethics approval and consent to participate

This study was approved by the institutional ethics committee of our hospital (No. 2016-0122).

## Results

### Clinical findings

In total, there were 30 males and 26 females, with a mean age of 35.2 years at surgery (range, 10–74 years). Cohort 1 and cohort 2 included 30 and 26 patients, respectively. The histological diagnoses for overall cohorts were enchondroma in 28 patients, osteoid osteoma, non-ossifying fibroma, and solitary bone cyst in 4 each, chondroblastoma in 3, aneurysmal bone cyst, giant cell tumor of bone, fibrous dysplasia, intraosseous lipoma, intraosseous ganglion, and intraosseous hemangioma in 1 each, and 7 in others. The affected bones were the tibia (n = 12), femur (n = 4), radius (n = 3), fibula (n = 3), calcaneus (n = 3), patella (n = 2), humerus (n = 2), and acetabulum (n = 1) in cohort 1 and phalanx of finger or toe (n = 18) and metacarpal bone (n = 8) in cohort 2. Among 24 cases of long bones in cohort 1, the locations within bone were an epiphysis in 5, metaphysis in 9, and diaphysis in 10. Demographic data of the cohort 1 and cohort 2 patients are shown in Tables 1 and 2, respectively. No complications, including infection, postoperative fracture, and allergic reaction, occurred related to the use of Affinos. No patients received postoperative treatments that affected bone metabolisms, such as bone modifying agents, steroid, cancer treatment, radiation, or long-term immobilization during this study.

**Table 1:** Demographic data of 30 patients in cohort 1.

Characteristics	Values or no. of patients
**Age, years**	
Mean (range)	29.7 (10–75)
**Gender**	
Female	9
Male	21
**Anatomic location**	
Tibia	12
Femur	4
Radius	3
Fibula	3
Calcaneus	3
Patella	2
Humerus	2
Acetabulum	1
**Defect volume, cm**^**3**^	
Mean (range)	16.1 (0.3–86)
**Histological diagnosis**	
Solitary bone cyst	4
Enchondroma	4
Non-ossifying fibroma	4
Osteoid ostoma	4
Chondroblastoma	2
Others	12
**Location within the bone (n = 24)**	
Diaphysis	10
Metaphysis	9
Epiphysis	5

**Table 2:** Demographic data of 26 patients in cohort 2.

Characteristics	Values or no. of patients
**Age, years**	
Mean (range)	42.1 (22–73)
**Gender**	
Female	9
Male	17
**Anatomic location**	
Phalanges of finger	13
Phalanges of toe	5
Metacarpal bones	8
**Defect volume, cm**^**3**^	
Mean (range)	1.5 (0.3–10)
**Histological diagnosis**	
Enchondroma	24
Chondroblastoma	1
Solitary bone cyst	1

### Radiographic findings

**Figure 3:**
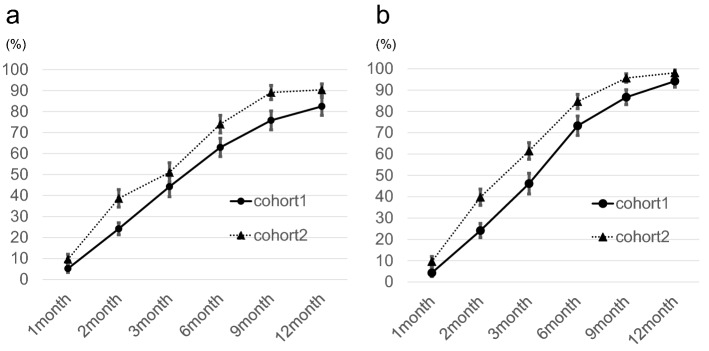
Results of evaluation with plain radiographs during the follow-up period. (**a**) The values of material resorption. (**b**) The values of trabeculation through the defect. The results are presented as the mean, and the bars indicate standard errors.

The mean volume of the bone defect immediately after surgery was 16.1 (range, 0.3–86) cm^3^ in cohort 1 and 1.5 cm^3^ (range, 0.3–10) in cohort 2. Material resorption and trabeculation through the defect were assessed with plain radiographs. The mean values of resorption and trabeculation increased steadily with time, and trabeculation progressed compared with resorption at each time point (Fig. 3a and b). The values of resorption and trabeculation 12 months after surgery were 82.5% (range, 25–100) and 94.2% (range, 25–100) in cohort 1 and 89.0% (range, 75–100) and 98.1% (range, 75–100) in cohort 2, respectively. Repeated measures ANOVA revealed that the values of trabeculation of cohort 2 were significantly increased with those of cohort 1 (p = 0.019), and Affinos tended to be resorbed in cohort 2 compared with cohort 1 (p = 0.076). Kappa analysis showed a high degree of agreement between investigators for material resorption (κ = 0.82) and trabeculation through the defect (κ = 0.88).

### Variables affecting the healing status of the bone defect

In cohort 1, multiple regression analyses showed that the location of the diaphysis, smaller defect volume, and the increased values of resorption 3 months postoperatively were associated with the increased value of resorption 12 months after surgery (R^2^ = 0.66, p < 0.001). The only variable associated with the values of trabeculation 12 months after surgery was the trabeculation value 2 months after surgery (R^2^ = 0.35, p = 0.002). No clinical variables were associated with the values of trabeculation 12 months after surgery in cohort 1.

In cohort 2, the increased values of resorption 2 months after surgery and smaller defect volume significantly correlated with the increased values of resorption 12 months after surgery (R^2^ = 0.58, p < 0.001). Multiple regression analysis revealed no variables related to the values of trabeculation 12 months after surgery.

### CT evaluation

To validate scores evaluated with plain radiographs, CT images were taken 3 and 9 months after surgery in 48 patients. Cohort 1 included 27 patients, and cohort 2 21. CT was not performed at each visit due to cost and radiation exposure. We analyzed whether there was any difference between the radiographic and CT findings at the same time. No significant differences were detected in the values of resorption or trabeculation between plain radiographs and CT images 3 months after surgery. At 9 months after surgery, the values of trabeculation were significantly lower in CT than in plain radiographic evaluation (p = 0.014). There was no significant difference in the values of resorption between plain radiographs and CT images 9 months after surgery.

### Bone metabolic markers

**Figure 4:**
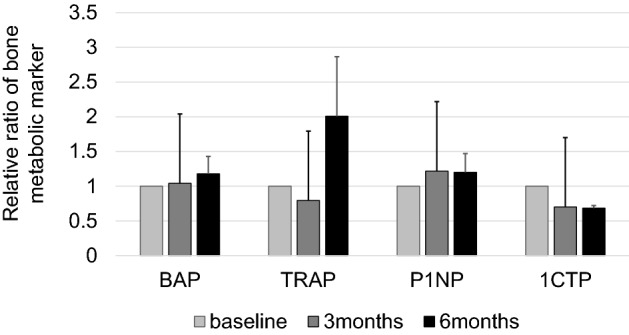
The relative ratio of bone metabolic markers obtained from 12 patients the next day (baseline), 3 months, and 6 months after surgery. The results are presented as the mean, and the bars indicate standard errors.

Three of the 15 patients were excluded from the analysis because they were missing any of the blood test items. Consecutive data were obtained from 12 patients. Demographic and clinical characteristics are shown in Table 3. Due to the small number of patients, we could not analyze the data by cohorts. The relative ratio from the baseline of bone metabolic markers was used for the analysis (Fig. 4). Spearman rank method identified a negative correlation between the ratio of 1CTP 3 months after surgery and the values of trabeculation 12 months after surgery (R = − 0.791, p = 0.004). In addition, the ratio of 1CTP 3 months after surgery tended to relate to the values of resorption 12 months after surgery (R = − 0.541, p = 0.086). No significant correlations were found between other markers (BAP, P1NP, and TRACP-5b) and the healing status.

**Table 3:** Demographic data of 12 patients evaluated bone metabolic markers with serum examination.

Variables	Values
**Age, years**	
Mean (range)	34.4 (13–56)
**Gender**	
Female	3
Male	9
**Anatomic location**	
Tibia	5
Radius	1
Fibula	1
Patella	1
Humerus	1
Phalanges	2
Metacarpal bone	1
**Defect volume, cm**^**3**^	
Mean (range)	10.4 (0.4–36.8)
**Histological diagnosis**	
Enchondroma	3
Fibrous dysplasia	1
Non-ossifying fibroma	2
Osteoid ostoma	2
Chondroblastoma	2
Others	2

## Discussion

An increasing number of synthetic bone substitutes have become commercially available as alternatives to autologous and allogenic bone grafts. Various studies have reported favorable outcomes with the use of β-TCP for bone defects. Despite only a small number of case reports being available, Affinos has been reported to be used successfully in clinical cases^11–13^. The present study also showed that Affinos maintains a balance between material resorption and trabeculation through the defect, resulting in stable clinical outcomes. When the rate of material resorption becomes faster than that of trabeculation through the defect, its mechanical strength is weakened, and it becomes ineffective as a scaffold for the articular loading surface. Although curettage without grafting has been performed in a clinical setting, this may raise concerns about the healing quality and healing time^14^. Hirn et al.^15^ reported a significant incidence of postoperative fractures and secondary osteoarthritis changes in defects larger than 60 cm^3^ without bone graft supplementation around the knee.

This study found that the values of bone remodeling with radiographic evaluation 2–3 months after surgery could reflect the healing status 12 months after surgery in both cohorts. Previous studies have rarely discussed the relationship between the degree of remodeling within bone defects during the follow-up period and bone healing after surgery. In the present study, defect volume was also detected as an independent factor associated with material resorption 12 months after surgery in both cohorts. Nicholas et al.^16^ reported that the healing of defects after curettage depended on the defect volume. A recent study with CT-based analysis showed a significant negative correlation between defect volume and the ratio of incorporated bone to defect volume^17^. Galois et al.^18^ mentioned that defect volume and age were significant factors affecting the incorporation of the material into the host bone. These results were in line with the findings in the present study. Older age was not an independently significant factor in our regression model.

Histological findings in animal models revealed that a unidirectional porous structure had an advantage in angiogenesis within materials^4,5^. This finding supported the theoretical merit of Affinos to promote material remodeling compared to β-TCP with a spherical interconnected porous structure^5,12^. Recently, a retrospective study comparing the regeneration of different β-TCP materials in high tibial osteotomies showed that bone remodeling progressed earlier with Affinos than with spherical porous β-TCP with a porosity of 60%, which was equivalent to that of Affinos^17^. In the present study, we examined the ability of a unidirectional porous β-TCP to regenerate bone in patients treated with resection of benign bone lesions followed by filling with Affinos. The evaluation with radiographic parameters showed that the values of trabeculation through the defects preceded those of material resorption over time. Although we did not conduct a randomized controlled trial with comparison to other bone substitutes, the result of our study suggested that the balance between trabeculation and material resorption of Affinos was beneficial for the treatment of bony defects after resection of benign bone lesions.

To date, various bone metabolic markers specific to the clinical monitoring of fracture healing have been investigated^7,8^. However, bone metabolic markers as surrogates of the activity of bone remodeling may provide additional information in addition to radiographic assessments. 1CTP is a bone resorption marker, which significantly increases in patients with bone metastasis and has a prognostic value for a progression of bone metastasis^19^. In our study, the low ratio of 1CTP at 3 months postoperatively showed a correlation with the healing status of bone defects in patients treated with Affinos. It would be advantageous for the repair of bone defects that the resorption of the surrounding bone subsides early after the surgery. However, our analysis to measure bone metabolic markers had low statistical power, and included only 12 patients, and so caution is needed to interpret the results. These data do not support the routine measurement of any metabolic markers, including 1CTP, to assess the progression of the healing process. Further research is warranted to assess their potential in monitoring the healing process.

CT images can depict trabecular patterns more accurately than plain radiographs. In this study, a statistical difference between evaluation with CT images and plain radiographs was observed only in the values of trabeculation 9 months after surgery in cohort 1. Using the scoring in this study, plain radiographs may be sufficient for the assessment of early postoperative status for the bone defect followed by filling bone substitutes.

This study has several limitations. First, it focused on only a small number of patients. Second, the histology was heterogeneous and included lesions with diverse biological behaviors, and so comparing the regeneration process of Affinos in the defects may not be completely accurate. Third, we did not have concurrent controls and did not compare our outcome with that of use of any other materials. Fourth, granules may not reflect the same mechanical strength as blocks. Granules are manufactured by classifying fired objects into the prescribed sizes and theoretically have the same internal structure as blocks. However, the mechanical strength of granules differs from that of blocks because the unidirectional porous structure of granules cannot be aligned in the defects. Finally, no functional or mechanical analyses were conducted. Thus, there is still a need for prospective randomized studies to evaluate the effect of different bone substitutes on the remodeling of the defects.

## Conclusions

The present study also showed the usefulness of low-porous β-TCP with a unidirectional porous structure for filling the defects after surgery for benign bone lesions. A combination of evaluation with radiographs and bone metabolism markers at an early stage postoperatively may help clinicians predict the clinical course of patients. Our results need to be confirmed in larger sample sizes and longer follow-up periods.

## Data Availability

The datasets used and analyzed during this study are available from the corresponding author (KI) upon reasonable request.
